# 8-O-Acetyl Shanzhiside Methylester From Lamiophlomis Rotata Reduces Neuropathic Pain by Inhibiting the ERK/TNF-α Pathway in Spinal Astrocytes

**DOI:** 10.3389/fncel.2018.00054

**Published:** 2018-03-08

**Authors:** Wei Zhang, Yang Bai, Yu Qiao, Jian Wang, Meng-Ying Li, Jing-Wen Wang, Na Jia, Tao Chen, Yun-Qing Li, Ai-Dong Wen

**Affiliations:** ^1^Department of Pharmacy, Xijing Hospital, The Fourth Military Medical University, Xi’an, China; ^2^Department of Anatomy and K. K. Leung Brain Research Centre, The Fourth Military Medical University, Xi’an, China; ^3^Student Brigade, The Fourth Military Medical University, Xi’an, China; ^4^Department of Internal Medicine, 94750 Army Hospital of PLA, Longyan, China; ^5^Department of Endocrinology, Xijing Hospital, The Fourth Military Medical University, Xi’an, China; ^6^Collaborative Innovation Center for Brain Science, Fudan University, Shanghai, China

**Keywords:** neuropathic pain, 8-O-acetyl shanzhiside methylester, ERK, SNL, spinal dorsal horn, astrocytes

## Abstract

*Lamiophlomis rotata* (*L. rotata*; Benth.) Kudo is an effective traditional herb in the clinical treatment of chronic pain syndromes in China. 8-O-acetyl shanzhiside methylester (8-OaS), a chief component in *L. rotata*, possesses potent immunosuppressive activities and favorable analgesic effects. This study was proposed to compare the analgesic effects of 8-OaS with those of lidocaine and ketamine in a spinal nerve ligation (SNL) model by behavioral tests, and then investigated its effects upon the expression of spinal glial fibrillary acidic protein (GFAP), phosphorylated extracellular regulated protein kinases (pERK) and tumor necrosis factor-alpha (TNF-α) via immunofluorescence staining and western blot analyses. The data showed consecutive intrathecal injection of 8-OaS for 2 weeks brought about remarkable palliation of neuropathic pain (NP), possessing similar anti-allodynia effects with those of lidocaine and ketamine. Two weeks after surgery, pERK within the spinal dorsal horn was mainly expressed in astrocytes more than neurons and microglia, and 8-OaS inhibited spinal astrocytic activation and TNF-α expression. Finally, co-treatment of 8-OaS and PD98059 (an Extracellular signal-regulated kinase, ERK inhibitor) did not lead to remarkable increase in pain relief or TNF-α expression comparing to rats treated with 8-OaS or PD98059 alone. In conclusion, the anti-nociceptive effects of 8-OaS in the condition of NP relied on the inhibition of SNL-induced astrocyte activation, probably via the down-regulation of the ERK/TNF-α pathway.

## Introduction

Neuropathic pain (NP) remains a common clinical syndrome that affects human well-being all over the world. The lack of effective drugs brings about a huge challenge in the field of NP treatment (Baron et al., 2010; Finnerup et al., 2015). Mounting evidence has implicated that spinal glial cells are essential in producing and maintaining NP (Zhuang et al., 2005; Milligan and Watkins, 2009). In the condition of NP, glial cells are activated via neuronal-glial interactions and then various pronociceptive mediators, such as cytokines (interleukin-1 beta, IL-1β and tumor necrosis factor-alpha, TNF-α) and neurotrophic factors, are produced to enhance neuronal activity via glial-neuronal interactions (Basbaum et al., 2009; Ji et al., 2013, 2016). Extracellular signal-regulated kinase (ERK), p38 and c-Jun N-terminal kinase (JNK) consist of the mitogen-activated protein kinase (MAPK) family within the spinal glia and play a pivotal role in the chronification of NP (Ji et al., 2009). As a canonical signaling molecule, ERK is activated by nerve injury in microglia and astrocytes sequentially and links the glial activation and the generation of inflammatory factors (Ji and Strichartz, 2004). Therefore, targeting spinal neuroinflammation processes renders novel therapeutic hopes for the treatment of NP (Ji et al., 2014).

Recent years have witnessed the prosperity of alternative therapy in the domain of clinical pain control. *Lamiophlomis rotata* (*L. rotata*) Kudo (Duyiwei in Chinese) is a traditional Tibetan herb for the remedy of knife wounds and various chronic pain syndromes for centuries, which was brought into the 2005 edition of Chinese Pharmacopoeia (State Pharmacopoeia Commission of the PRC, 2005). The active ingredients of the analgesic are the iridoid glycosides of *L. rotata* (IGLR), mainly the shanzhiside methylester (SM) and 8-O-acetyl-SM (8-OaS; Yi et al., 1997; Shang et al., 2011; La et al., 2015). Emerging evidence has suggested that spinal neuroinflammatory mechanisms play a pivotal role in IGLR-induced analgesia. IGLR relieves chronic NP probably by the suppression of neuroinflammation in the spinal cord (Zheng et al., 2015). During this process, spinal microglia are recruited via the p38 signaling pathway to facilitate β-endorphin production to ameliorate NP (Fan et al., 2016). However, it is not well-known yet whether spinal astrocytes are involved in IGLR-induced pain relief.

This study aimed to explore the involvement of astrocytic inflammatory mechanisms in 8-OaS induced analgesia a spinal nerve ligation (SNL) rat model. First, the anti-nociceptive effects of 8-OaS were compared to those of lidocaine and ketamine. Then, the activation of astrocytes, the function of the ERK pathway as well as the expression of TNF-α were sequentially examined following 8-OaS administration. Finally, an ERK inhibitor was also co-administered to probe into the possible signaling pathway underlying the analgesic effects of 8-OaS.

## Materials and Methods

### Animals, Drugs and Experimental Design

Male Sprague-Dawley rats (220–250 g) were utilized in the present study. The rats were housed on an 12-h light-dark cycle. All the experiments in the present study were performed according to the ethical guidelines of the International Association for the Study of Pain and approved by The First Affiliated Hospital of Fourth Military Medical University Committee on Animal Care and Use. All efforts were made to minimize the number of animals used and animal suffering.

Drugs used in this study were as follows. 8-OaS, provided by Shandong Engineering Research Center for Nature Drug, China, was diluted in 0.9% saline containing 10% dimethyl sulfoxide (DMSO) to concentrations of 5, 10, 20 and 40 μg/10 μl, respectively. Lidocaine, a common local anesthetic in clinical practice, was diluted with 0.9% saline to the concentration of 100 μg/10 μl. Each rat was given 100 μg according to previous studies (Ma et al., 2003; Cheng et al., 2011). Ketamine, a noncompetitive N-methyl-D-aspartate receptor (NMDAR) antagonist for treating NP (Kozek et al., 2006), was diluted with 0.9% saline to the concentration of 10 μg/10 μl. Each rat was given 25 μg according to our previous study (Mei et al., 2011). PD98059, a specific ERK1/2 inhibitor, was dissolved in 0.9% saline containing 10% DMSO to the concentration of 10 μg/10 μl. Each rat was given 10 μg according to previous studies (Zhuang et al., 2005; van den Heuvel et al., 2015).

The experimental design was as follows. First, to verify the analgesic effects of 8-OaS in NP, 56 rats were divided into seven groups in the experiments: normal group, sham-saline group, SNL-saline group, SNL-8-OaS (5 μg) group, SNL-8-OaS (10 μg) group, SNL-8-OaS (20 μg) group, SNL-8-OaS (40 μg) group (*n* = 8 in each group). Rats were given different doses (5, 10, 20 and 40 μg) 8-OaS or saline from post-operation days (POD) 2 to 15 everyday and nociceptive behavioral tests were performed ahead of SNL (baseline), and on POD 1–22 every other day.

Second, to compare the antinociceptive effects of 8-OaS with those of ketamine and lidocaine, 30 rats were divided into five groups: sham-saline group, SNL-saline group, SNL-lidocaine (100 μg) group, SNL-ketamine (20 μg) group, SNL-8-OaS (20 μg) group (*n* = 6 in each group). Drugs or saline were injected from POD 2 to 8 everyday and nociceptive behavioral tests were performed ahead of SNL (baseline), and on POD 1–14 every day.

Third, to investigate the activation of astrocytes and ERK as well as the production of TNF-α after nerve injury, 16 rats were divided into four groups: sham group, SNL-3d group, SNL-7d group and SNL-15d group. The spinal cord was harvested according to the time points and western blot analyses were performed.

Next, to explore the effects of 8-OaS on the expression of spinal glial fibrillary acidic protein (GFAP), phosphorylated extracellular regulated protein kinases (pERK) and TNF-α, 28 rats were divided into four groups: sham group, sham-8-OaS group, SNL-saline group and SNL-8-OaS (20 μg) group (*n* = 7 in each group, four rats for western blot and three for immunostaining analyses). Drugs or saline were given from POD 2 to 15 everyday and the spinal cords were harvested on POD 15 for western blot and immunostaining analyses.

Finally, to further verify the role of ERK in the analgesic effects of 8-OaS, 24 rats were divided into four groups: SNL-saline group, SNL-PD98059 (10 μg) group, SNL-8-OaS (20 μg) group, SNL-PD98059 (10 μg) + 8-OaS (20 μg) group (*n* = 6 in each group). The rats were used for behavioral tests and then the spinal cords were harvested for western blot on POD 15. Drugs or saline were injected from POD 2 to 15 everyday and nociceptive behavioral tests were performed ahead of SNL (baseline), and on POD 1–15 every 3 days.

### Spinal Nerve Ligation Model

SNL model was performed according to our previous study (Wang W. et al., 2012). First, the rats were anesthetized with intraperitoneal injection of chloral hydrate (280 mg/kg). Next, the transverse process of the sixth left lumbar was cut off to expose the L4 and L5 spinal nerves. Subsequently, the L5 spinal nerve was separated and ligated by 6–0 silk sutures, and the incision was closed layer by layer. Rats in the sham group received the same procedures except for nerve ligation.

### Drug Administration

For the implantation of the intrathecal catheter, the animal was deeply anesthetized and laminectomy was carried out on the thoracic spine. A polyethylene drain was inserted to the spinal cord, ranging from the L4 to L5 level. Subsequently, 200 μg lidocaine (in 10 μl) was injected to the rats via the intrathecal catheter to judge the proper location of the PE tubing by positive signs that the hind legs were completely paralyzed following lidocaine administration. Drugs and saline were injected intrathecally over 30 s, followed by a 10 μl flush of normal saline. Two hours after drug administration, nociceptive behavioral tests were performed.

### Nociceptive Behavioral Tests

The rats were acclimated to the experiment room 72 h prior to the preliminary tests. They then stayed inside plastic boxes upon an elevated mesh floor and were allowed to habituate for 30 min ahead of the threshold tests. Mechanical thresholds were examined using von Frey filaments. The ipsilateral hind paw was pressed by a series of von Frey filaments that steadily increased in stiffness (2, 4, 6, 8, 10, 15 and 26 g), each for 5–6 s for each filament with 10 s interval. The minimal force causing withdrawn responses at least 6 times in 10 stimulations was considered as the paw withdrawal threshold (PWT). Positive signs for withdrawal behaviors included neat withdrawal, nibbling, ipsilateral rear leg vibrating as well as producing sound.

### Double Immunofluorescence Staining

On POD 15, the anesthetized rats were transcardially perfused with the 100 ml of 0.01 M phosphate buffer saline (PBS, pH 7.3) and then 500 ml of 0.1 M phosphate buffer (PB, pH 7.3) containing 4% paraformaldehyde. Next, the L5 segments of the spinal cord were harvested according to the termination of the L4 and L5 dorsal roots, and subsequently immersed in 0.1 M PB containing 30% sucrose at 4°C. Transverse spinal segments with a thickness of 25 μm were cut on a cryostat (Leica CM1800; Heidelberg, Germany) and then performed for immunohistochemistry in accordance with our previous study. For immunofluorescence staining, these slices were rinsed for three times (each for 10 min) within 0.01 M PBS and then placed in 2% goat serum blocking solution (diluted with 0.01 M PBS) containing 0.3% Triton X-100 for 1 h at room temperature (RT). Then, the sections were incubated with following primary antibodies overnight at 4°C: rabbit anti-pERK (1:500; Cell Signalling Technology, MA, USA) together with mouse anti-GFAP (1:500; Millipore, MA, USA) or goat anti-Iba-1 (1:500; Millipore) or mouse anti-NeuN (1:500; Millipore). After three washes within 0.01 M PBS, these slices were then incubated with corresponding secondary antibodies for 4 h at RT: Alexa Fluor 594-conjugated donkey anti-rabbit IgG (1:1000; Molecular Probes, OR, USA) and Alexa Fluor 488-conjugated donkey anti-mouse or goat IgG (1:1000; Molecular Probes). A confocal laser microscope (FV1000; Olympus, Tokyo, Japan) was used to observe digital images which were captured using FluoView 1000 (CLSM, FV1000; Olympus, Tokyo, Japan). Around 15 slices obtained from three rats (five slices per rat) were randomly chosen. Images were carried out by individuals blinded towards the experimental groups.

### Western Blot Analysis

The animals were anesthetized with 7% chloral hydrate (280 mg/kg, i.p.) and rapidly sacrificed. The dorsal portion of the L5 segments were harvested on ice and then homogenized in sodium dodecyl sulfate (SDS) sample buffer (10 ml/mg tissue) containing proteinase and phosphatase inhibitors (Sigma-Aldrich, MO, USA). Subsequently, 10 μg protein from each sample (quantitatively measured by the BCA protein assay; Thermo Scientific; Rockford, IL, USA) was subjected to 10% SDS-polyacrylamide gel (SDS-PAGE) electrophoresis and electrophoretically transferred to polyvinylidene difluoride (PVDF) membranes (Immobilon-P, Millipore, CA, USA). After blocking in PBS containing 5% DifcoTM skim milk for 2 h, the membranes were incubated overnight at 4°C with following primary antibodies: rabbit anti-pERK (1:1000; Cell Signalling Technology), rabbit anti-ERK (1:1000; Cell Signaling Technology), mouse anti-GFAP (1:5000; Millipore), goat anti-TNF-α (1:500; Millipore) and mouse anti-GAPDH (1:5000; Millipore). The primary antibodies were detected using horseradish peroxidase (HRP)-conjugated secondary antibodies (anti-rabbit, 1:3000; anti-mouse, 1:5000 and anti-goat, 1:3000; Amersham Pharmacia Biotech Inc., NJ, USA). The membranes were rinsed three times (10 min each) within TBST between each step. The enhanced chemiluminescence (ECL) detection method (Amersham) was used to detect all bands in the present study. The densities of the proteins on the blots were analyzed by LabWorks Software (Ultra-Violet Products, UK). The densities of the target proteins as well as the GAPDH-immunoreactive bands were quantified by subtracting the background. Squares of the same size were drawn around each band to measure the density and the background within the square and near the band was subtracted. The target protein levels were normalized to GAPDH levels and expressed as relative fold changes compared to the naïve or sham-vehicle group.

### Data Analyses

Data were presented as means ± standard errors of the means (SEM) and were analyzed by researchers who were blinded to the experimental design. Repeated-measure of analysis of variance (ANOVA) followed by LSD *post hoc* tests were used for multiple comparisons for consecutive nociceptive behavioral tests (SPSS 17.0). The Student’s *t* test (for comparisons between two groups) and one-way ANOVA followed by LSD *post hoc* tests (for comparisons among multiple groups) were used in the western blot analyses. The dose-response curve of 8-OaS was constructed by four doses with eight rats at each dose and the median effective dose (ED50) was calculated via the graded dose-response method. The values for the area under the time-course curves (AUCs) were calculated to measure the effects of different doses of 8-OaS. The analyzed numbers for each experiment were indicated in the corresponding figure legends. *P* < 0.05 was considered statistically significant.

## Results

### Consecutive Intrathecal Administration of 8-OaS Attenuated SNL-Induced Mechanical Hypersensitivity in a Dose-Dependent Manner

**Figure 1:**
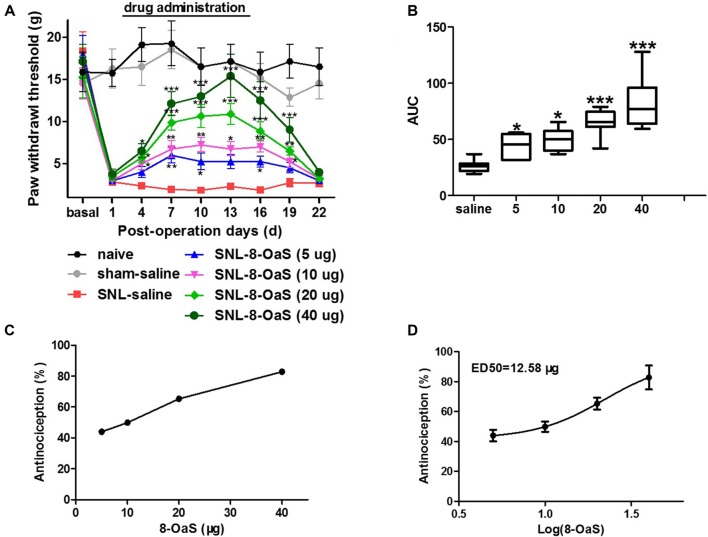
Effects of consecutive intrathecal administration of 8-O-acetyl shanzhiside methylester (8-OaS) on spinal nerve ligation (SNL)-induced mechanical hypersensitivity. The time course of analgesia by different doses of 8-OaS **(A)**. The area under the time-course curves (AUCs) of the analgesic effects for different doses of 8-OaS **(B)**. The dose effect or log (dose) effect curves for the analgesic effects of 8-OaS are shown in **(C,D)**, respectively. *P < 0.05, **P < 0.01 and ***P < 0.001, compared with the SNL-saline group. *n* = 8 in each group.

The effects of 8-OaS administration from POD 2 to 15 on mechanical hypersensitivity in the SNL model were evaluated from POD 1 to 22. As shown in the Figure 1A, SNL led to significant mechanical hypersensitivity compared to the sham-saline group. Low dose 8-OaS (5 μg) reversed SNL-induced mechanical hypersensitivity from POD 7 to 16, while high doses (10 μg, 20 μg and 40 μg) exhibited higher and longer analgesic effects from POD 4 to 19 (Figures 1A,B). Furthermore, the effect of 8-OaS on SNL-induced PWL changes was counted in accordance with the dose-response curve (Figure 1C), through which ED50 were calculated (Figure 1D). The ED50 for 8-OaS on SNL-induced mechanical allodynia was 12.58 μg, which paved the way for the selection of drug dose in the comparison of analgesic effects among 8-OaS, lidocaine and ketamine.

### The Comparison of the Anti-nociceptive Effects of Lidocaine, Ketamine and 8-OaS on SNL-Induced Mechanical Hypersensitivity

**Figure 2:**
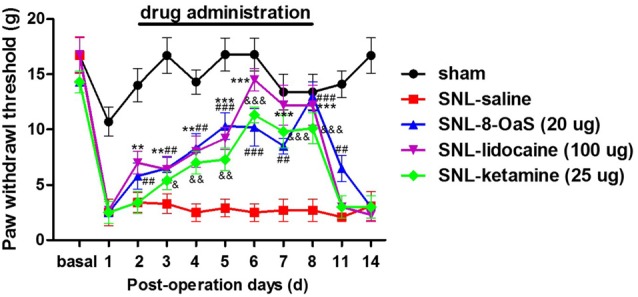
The analgesic effects of lidocaine, ketamine and 8-OaS in the SNL model. Treatments with lidocaine, ketamine and 8-OaS for consecutive 7 days decreased SNL-induced mechanical hypersensitivity, **P < 0.01 and ***P < 0.001, SNL-lidocaine vs. SNL-saline group; ^&^*P* < 0.05, ^&&^*P* < 0.01 and ^&&&^*P* < 0.001, SNL-ketamine vs. SNL-saline group; ^##^*P* < 0.01 and ^###^*P* < 0.001, SNL-8-OaS vs. SNL-saline group. *n* = 6 in each group.

We selected lidocaine and ketamine as positive control to evaluate the analgesic effects of 8-OaS (20 μg) on rats with NP. The doses of lidocaine (100 μg) and ketamine (25 μg) have been reported to exhibit favorable analgesic effects in previous studies (Ma et al., 2003; Kozek et al., 2006). As shown in the behavioral tests, intrathetical injection (from POD 2 to 8) of lidocaine, ketamine and 8-OaS significantly attenuated SNL-induced mechanical allodynia (Figure 2). Lidocaine and 8-OaS had immediate anti-nociceptive effects on POD 2 (*P* < 0.05, SNL-lidocaine or 8-OaS vs. SNL-vehicle group), while ketamine took effects on POD 3. On POD 6, the anti-nociceptive effects of lidocaine and ketamine reached the peak and lidocaine exhibited more favorable analgesic effects that the other two (*P* < 0.001, SNL-lidocaine vs. SNL-ketamine or 8-OaS group). However, on POD 8, the analgesic effects of 8-OaS got to its climax and there was no significant difference between the maximum analgesic effects of 8-OaS and lidocaine on POD 8 and 6, respectively (*P* > 0.05, SNL-8-OaS on POD 8 vs. SNL-lidocaine on POD 8 or SNL-lidocaine on POD 6). Interestingly, on POD 11 (3 days after the last drug administration), 8-OaS still exerted anti-hypersensitivity effects (*P* < 0.05, SNL-8-OaS vs. SNL-vehicle group) while those of lidocaine and ketamine evaded (*P* > 0.05, SNL-lidocaine or ketamine vs. SNL-vehicle group). Generally, 8-OaS at the dose of 20 μg possessed equal but longer analgesic effects comparing to those of lidocaine and ketamine.

### SNL-Induced Increased ERK Activation in Astrocytes at POD 14

**Figure 3:**
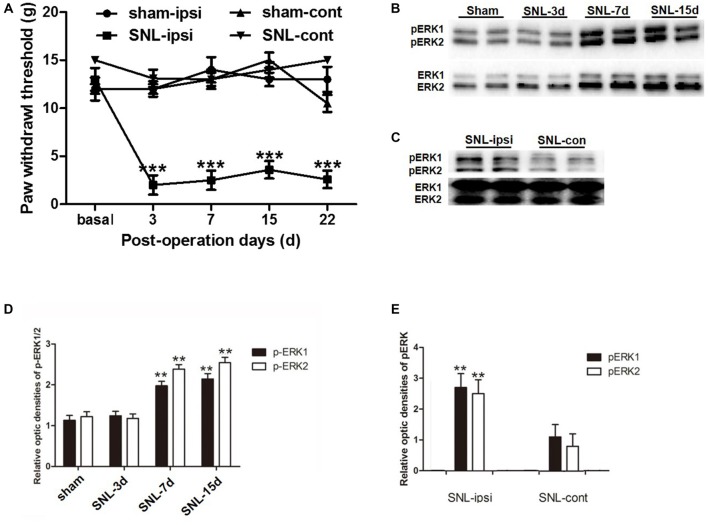
The up-regulation of phosphorylated extracellular regulated protein kinases 1/2 (pERK1/2) within the spinal dorsal horn after SNL. SNL induced long-lasting ipsilateral mechanical hypersensitivity instead of contralateral hypersensitivity **(A)**. ***P < 0.001 compared with sham group. *n* = 6 in each group. Western blot analyses showed that SNL enhanced the level of spinal pERK1/2 from post-operation days (POD) 3 to POD 15 **(B,D)**. **P < 0.01 compared with sham group. The pERK1/2 in the SNL model was primarily expressed in the ipsilateral spinal dorsal horn instead of the contralateral part on POD 15 **(C,E)**. **P < 0.01 compared with the contralateral side. Duplication was introduced in the western blot analyses for accuracy judgment. *n* = 4 in each group.

In order to verify whether SNL-induced NP was accompanied with astrocytic ERK activation, we investigated astrocyte activation (indicated by GFAP expression) and ERK activation (indicated by its phosphorylation) in the spinal dorsal horn. According to behavioral tests, SNL induced ipsilateral mechanical hypersensitivity instead of contralateral hypersensitivity, which lasted until POD 22 (Figure 3A). Western blot analyses showed the expression of GFAP was significantly increased in the spinal dorsal horn on POD 15 after SNL in comparison to that of the sham group (*P* < 0.05; Figures 5A,C), which was corroborated by the enhanced staining of GFAP in SNL group (Figures 6A1,C1). All these data were in accordance with previous studies suggesting the proliferation of astrocytes in various NP models (Garrison et al., 1991; Tsuda et al., 2011).

**Figure 4:**
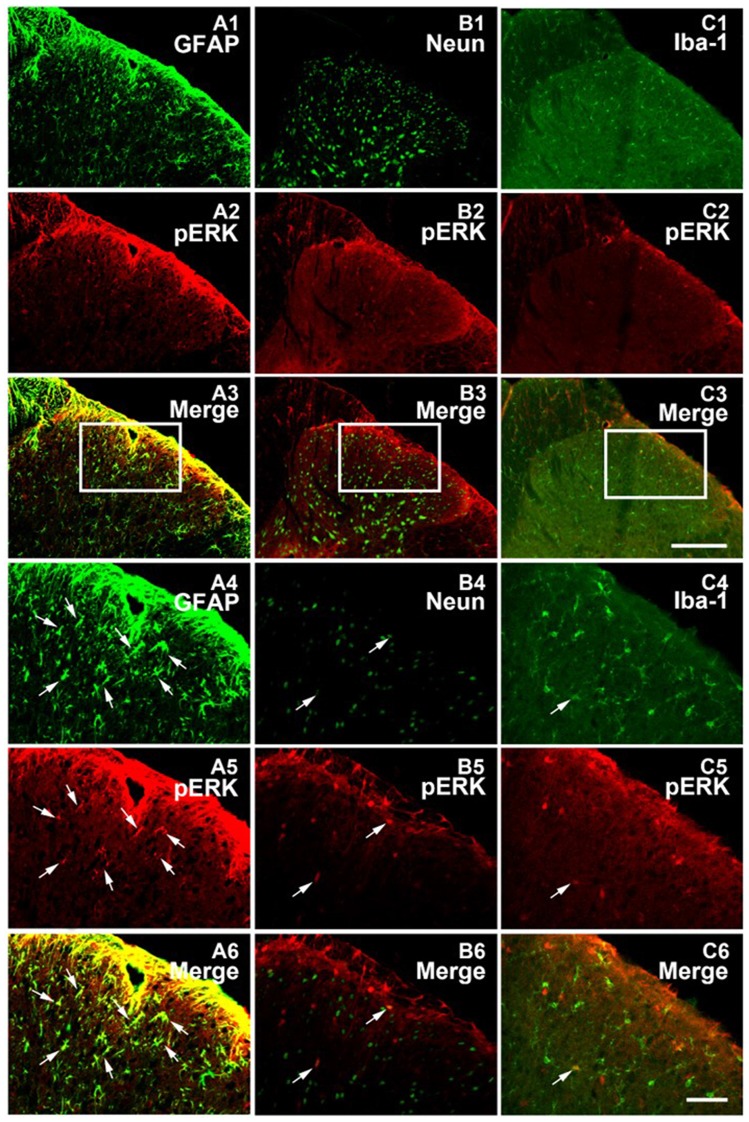
pERK was primarily expressed within spinal astrocytes in the SNL model on POD 15. Microphotographs indicating double-immunofluorescence histochemistry for pERK (red) and glial fibrillary acidic protein (GFAP; **A1–A6**) or NeuN **(B1–B6)** or Iba-1 (**C1–C6**; green) immunoreactivities within ipsilateral spinal dorsal horn in the SNL model on POD15. The framed areas in images **(A1–A3,B1–B3,C1–C3)** were magnified in images **(A4–A6, B4–B6, C4–C6)**, respectively. White arrows showed double-labeled neurons in each set pictures. Bars = 100 μm in images **(A1–A3,B1–B3,C1–C3)** and 40 μm in images **(A4–A6,B4–B6,C4–C6)**.

**Figure 5:**
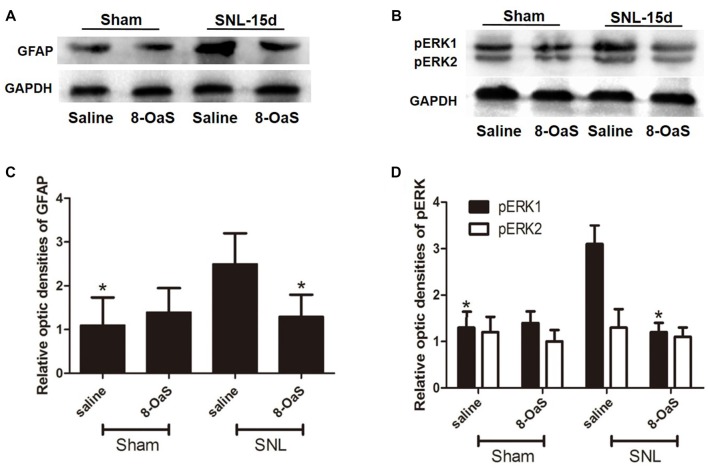
The effects of 8-OaS administration on SNL-induced spinal GFAP and pERK upregulation. Western blot analysis showed that GFAP **(A,C)** and pERK **(B,D)** expression increased in the ipsilateral spinal dorsal horn of SNL rats at POD 15. 8-OaS reduced the expression of GFAP in the SNL model **(A,C)**. 8-OaS also suppressed SNL-induced pERK1 but not pERK2 **(B,D)** expression. *P < 0.05 compared with saline-SNL group. *n* = 4 for each group.

**Figure 6:**
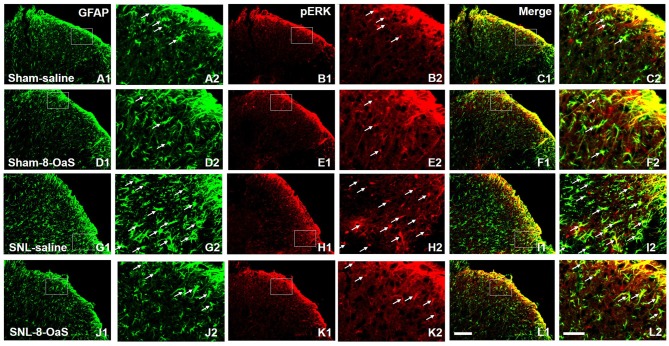
8-OaS inhibited SNL-induced spinal astrocytic ERK activation. SNL induced a marked increase in astrocytic activation and ERK activation in the ipsilateral spinal dorsal horn on POD 15, as indicated by the up-regulation of GFAP staining **(A,G)** and pERK staining **(B,H)** respectively. The GFAP/pERK double-labeled neurons **(C,I)** were also increased after SNL. 8-OaS decreased the density of GFAP **(G,J)** and pERK **(H,K)** staining as well as the number of GFAP and pERK double-labeled neurons **(I,L)** in the ipsilateral spinal dorsal horn after SNL. The framed area in images **(A–L)** was magnified in images **(A′–L′)**, respectively. White arrows showed double-labeled neurons in each set of pictures. Bars = 100 μm in images **(A1–L1)** and 40 μm in images **(A2–L2)**.

Previous studies suggested that in the condition of NP, pERK was expressed both in neurons and glial cells, but there was a transition of expression profile from microglia to astrocytes in different phases (Zhuang et al., 2005; Hald et al., 2009). First, we also observed an increase in spinal ERK (pERK1 and pERK2) phosphorylation from POD 7 to POD 14 (*P* < 0.01; in comparison with the sham group; Figures 3B,D), which predominated in the ipsilateral spinal dorsal horn rather than the contralateral part (*P* < 0.01; Figures 3C,E; Wang X. W. et al., 2012; van den Heuvel et al., 2015). Next, we examined the expression profile of pERK in the spinal dorsal horn on POD 15 in the SNL model. Double immunofluorescence staining showed that pERK was primarily colocalized with GFAP in the spinal dorsal horn rather than NeuN (a marker of neuron) and Iba-1 (a marker of microglia; Figure 4), suggesting that at this time point, pERK was mainly expressed in astrocytes. Taken together, these data indicated that SNL-induced increased ERK activation in astrocytes at POD 15.

### 8-OaS Inhibits SNL-Induced Increase in the Astrocytic pERK Activation

To explore the underlying mechanisms of 8-OaS analgesia, we further detected the effects of long-term administration of 8-OaS on astrocytic pERK activation after SNL on POD 15. Western blot analyses showed that on POD 15, 8-OaS remarkably decreased the expression of GFAP and pERK in the spinal dorsal horn in the SNL-saline group compared with that in the sham-saline group (*P* < 0.05; Figures 5A–D). However, 8-OaS did not affect GFAP or pERK expression in the sham-operated rats compared with that in the sham-saline group (*P* > 0.05; Figures 5A–D). Immunostaining analyses indicated 8-OaS clearly decreased the number of GFAP-ir and pERK-ir as well as double-labeled neurons within the spinal dorsal horn compared to the SNL-saline group (Figure 6), while no significant changes of GFAP and pERK expression between sham-8-OaS and sham-saline groups were observed (Figure 6), which was in accordance with the immunoblotting data. All of these results implied 8-OaS effectively inhibited astrocytic pERK up-regulation following SNL.

### 8-OaS Attenuates SNL-Induced Up-Regulation of TNF-α Via Inhibiting ERK Phosphorylation

**Figure 7:**
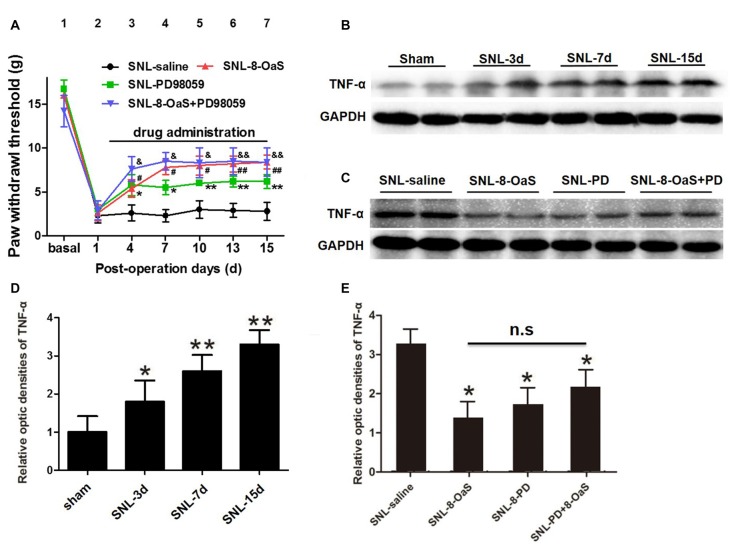
8-OaS suppressed SNL-induced spinal tumor necrosis factor-alpha (TNF-α) expression via inhibiting ERK activation. Both administration of 8-OaS and PD98059 increased mechanical paw withdrawal threshold (PWT) of the SNL model. But co-treatment of 8-OaS and PD98059 did not result in further significant increases in PWT compared to the 8-OaS group and the PD98059 group **(A)**. *P < 0.05, **P < 0.01, SNL-PD98095 group compared with SNL-saline group. ^#^*P* < 0.05, ^##^*P* < 0.01, SNL-8-OaS group compared with SNL-saline group. ^&^*P* < 0.05, ^&&^*P* < 0.01, SNL-8-OaS+PD98095 group compared with SNL-saline group. SNL surgery increased the expression of TNF-α within the ipsilateral spinal dorsal horn from POD 3 to 15 **(B,D)**. *P < 0.05, **P < 0.01, compared with the sham group. *n* = 4 in each group. The expression of TNF-α were inhibited in 8-OaS and PD98059 treated groups but co-treatment of 8-OaS and PD98059 did not lead to further inhibition of TNF-α **(C,E)**. *P < 0.05, compared with the SNL-saline group. *n* = 4 in each group. Duplication in the western blot analyses was introduced for accuracy judgment.

TNF-α is a crucial glial inflammatory mediator within the spinal dorsal horn in the condition of NP, which contributes to the induction of chronic pain (McMahon and Malcangio, 2009). In line with previous results (Xu et al., 2006; Zheng et al., 2011), overexpression of TNF-α was also observed in the condition of SNL-induced NP on from POD 3 to 15 (*P* < 0.05; Figures 7B,D). 8-OaS remarkably decreased the expression of spinal TNF-α in the SNL-8-OaS group compared with that in the SNL-saline group (*P* < 0.05; Figures 7C,E), which was consistent with an previous study (Zheng et al., 2015). These data implicated that 8-OaS possessed effective anti-inflammatory effects in the condition of NP.

To further verify whether the anti-inflammatory effects of 8-OaS analgesia were dependent on the inhibition of ERK pathway after SNL, PD98059 was intrathecally administered into the L4/5 spinal cord, and then mechanical PWT and spinal TNF-α expression were measured. In accordance with previous studies (Wang X. W. et al., 2012; van den Heuvel et al., 2015), PD98059 (10 μg) significantly elevated mechanical PWT compared with the vehicle control (*P* < 0.05; Figure 7A). However, co-treatment with 8-OaS and PD98059 failed to result in significant increase in mechanical PWT compared with the group treated with PD98059 or 8-OaS alone (*P* > 0.05; Figure 7A). Likewise, PD98059 alone significantly decreased the amount of spinal TNF-α, but co-treatment with 8-OaS and PD98059 did not result in further inhibition of TNF-α compared with the group treated with 8-OaS or PD98059 alone (*P* > 0.05; Figures 7B–E). Thus, the analgesic effect of 8-OaS possibly rely on the inhibition of ERK activation.

## Discussion

In the present study, we showed that 8-OaS, possessing equal but longer-lasting analgesic effects compared with lidocaine and ketamine, alleviated mechanical allodynia in a dose-dependent way in NP rats. In addition, the activity of spinal astrocytes and the ERK pathway were significantly inhibited. Furthermore, the release of TNF-α, a pro-inflammatory cytokine acting as the downstream signaling molecule of MAPK pathway, was significantly attenuated by intrathecal 8-OaS administration. Finally, co-administration of 8-OaS and an ERK inhibitor PD98059 did not lead to more pain relief and the inhibition of TNF-α expression compared to 8-OaS alone. Based on these results, the analgesic effect of 8-OaS was likely mediated by the inhibition of the ERK/TNF-α pathway in spinal astrocytes after SNL.

*L. rotata* is a traditional Chinese drug classified to phlomis umbrosa that grows in Tibetan Plateau. Many plants allied to phlomis umbrosa have been reported possessing anti-nociceptive and anti-inflammatory activities, which are mediated by the iridoid glycosides extracts as the main quality control ingredients. SM and 8-OaS, two principle iridoid glycosides in *L. rotata*, bear equal pharmacological potency and analgesic efficacy (Li et al., 2008; Tan et al., 2012; Zhu et al., 2014). These iridoid glycosides have been reported to alleviate formalin induced-tonic pain, acetic acid-induced writhing response, peripheral NP, cancer pain, traumatic pain and dysmenorrhea, instead of formalin-induced acute nociception and thermally-induced nociception (Shang et al., 2011; Zheng et al., 2015). In the present study, we characterized the anti-hypersensitivity effects of one principle ingredient of *L. ratata*, 8-OaS, in a rat model of SNL induced neuropathy. Consecutive intrathecal injection of 8-OaS dose-dependently reduced mechanical hypersensitivity of ipsilateral hind paw of neuropathic rats (ED50 = 12.58 μg) without anti-allodynic tolerance. Long-term intrathecal administration of 8-OaS (20 μg) exhibited equal but longer anti-hypersensitivity effects compared to lidocaine (100 μg) and ketamine (25 μg). Previous studies also verified that *L. rotata* did not generate anti-allodynic tolerance as well as locomotor activity disorder (Zhu et al., 2014; Zheng et al., 2015; Fan et al., 2016). All these merits suggested that *L. rotata* could be a validated drug for the treatment of chronic pain.

For a long time, *L. rotata* has been used as a strong painkiller and hemostatic in China, both with IGLR as the effective ingredients. It could exert effects via intraperitoneal injection at a high dose, with similar effects as non-steroidal anti-inflammatory drugs, that is, via inhibiting peritoneal capillary permeability, leukocyte infiltration and the release of endogenous mediators (Shang et al., 2011). In addition, intrathetical injection of *L. rotata* also produced favorable analgesic effects at a relative lower dose (Fan et al., 2016). The main mechanism of *L. rotata* analgesia within the central neural system is the regulation of spinal nociceptive message transmission (implicated by the inactivation of the NMDAR/PKC and NO/cGMP/PKG pathways) and spinal neuroinflammatory reactions (the decrease in TNF-α and IL-1β production and increase in IL-10 production; Zheng et al., 2015). The role of microglia has been clarified in this analgesic process. *L. ratata* binds to the GLP-1 receptor to facilitate microglia to secret β-endorphin, with the p38 MAPK signaling as a key linkage (Zhu et al., 2014; Fan et al., 2016). Our present study verified the role of astrocytes in *L. ratata* related analgesia, that is, the inhibition of astrocytic inflammatory reactions via ERK signaling pathways.

In the condition of nerve injury, activation of glial cells results in the development of neuroinflammation which is responsible for the induction and maintenance of chronic pain (Ji et al., 2013). This process is predominated by the early activation of microglia and subsequent long-term activation of astrocytes. Bearing receptors for various neurotransmitters released from nociceptive neurons and inflammatory factors from early-activated microglia, astrocytes respond to pain signals and then produce inflammatory factors including cytokines, prostaglandins and neurotrophic factors that in turn facilitate the transmission of pain signals within the spinal cord (Gao and Ji, 2010; Nakagawa and Kaneko, 2010). Thus, drugs inhibiting the activity of astrocytes brought about favorable chronic pain relief (Nakagawa and Kaneko, 2010; Ji et al., 2014). In the present study, we also observed the up-regulation of spinal GFAP expression in the condition of SNL and the alleviation of NP via 8-OaS was accompanied with the down-regulation of GFAP, suggesting that the analgesic effects of 8-OaS may possibly correlate with the inactivation of astrocytes.

There is a time sequence of ERK activation in different cell types within the spinal dorsal horn. After nerve injury, there is a transient ERK activation in neurons which release neurotransmitters to drive microglial ERK activation via neuronal-glial communications in the early phase of pathological pain (first several days). Then, astrocytic ERK is recruited by glial-glial interactions to sustain NP in the late phase (several weeks later; Ji et al., 2009). Nerve injury induced glial ERK activation is crucial for the intracellular signaling that leads to the activation of spinal glial cells, the production of proinflammatory cytokines, chemokines and growth factors, and then the sensitization of dorsal horn neurons (Ji et al., 2013). Accordingly, drugs targeting at ERK have been confirmed exerting desirable analgesic effects (Zhuang et al., 2005; Wang X. W. et al., 2012; Xu et al., 2013; van den Heuvel et al., 2015). Since our present study aimed at the role of astrocytes in 8-OaS-induced analgesia, long-term drug administration was adopted and the changes in the late phase of nerve injury were measured. In accordance with previous data (Zhuang et al., 2005), we observed that at POD15, ERK was mainly expressed in astrocytes. Meanwhile, 8-OaS analgesia was accompanied by the suppression of astrocytic ERK activation and ERK-selective inhibitor failed to lead to further pain relief with 8-OaS co-administration, suggesting that the analgesic effects of 8-OaS were probably mediated by the inhibition of spinal astrocytic ERK activity.

TNF-α is one representative proinflammatory cytokines produced by spinal astrocytes. TNF-α triggers central sensitization of chronic pain via postsynaptic recruitment of AMPAR and NMDAR in dorsal horn neurons (Ferguson et al., 2008; Choi et al., 2010). Drugs inhibiting the production of TNF-α and other pro-inflammatory cytokines have been proved to elicit satisfactory pain relief in NP (Xu et al., 2006; Zheng et al., 2011; Ji et al., 2014). In our study, the administration of 8-OaS also decreased the enhanced expression of TNF-α in the condition of chronic NP. Importantly, co-treatment of 8-OaS and PD98059 did not result in further down-regulation of spinal TNF-α production, further demonstrating that the analgesic effect of 8-OaS was associated with the inhibition of spinal inflammatory cascades via ERK-related pathway.

Apart from analgesic effects, 8-OaS has been reported to possessing neuro- and cardio-protective effects in the condition of oxygen and glucose deprivation by the suppression of inflammation and apoptosis-related cascades. What is worth noting is the improvement of mitochondrial energy metabolism (Jiang et al., 2010, 2011; Kang et al., 2012). Mitochondrial dysfunction is also implicated in the pathogenesis of NP, and protecting mitochondrial function is a promising strategy to alleviate or prevent chronic pain (Sui et al., 2013). In light of this, we proposed that the regulation of mitochondrial disorder and neuron apoptosis might be another mechanism for 8-OaS analgesia, which remains to be explored in future studies. These anti-inflammation and apoptosis merits may endow 8-OaS as a promising therapeutic candidate in the treatment of chronic pain and other diseases.

## Conclusion

Our present study suggested that 8-OaS had favorable analgesic effects on SNL-induced NP. The analgesic effects of 8-OaS may be owing to the anti-inflammatory effects within the spinal dorsal horn, that is, the inhibition of astrocytic ERK phosphorylation and subsequent TNF-α production. These data provide evidence for understanding the mechanisms underlying 8-OaS analgesia and support a promising orientation in the field of NP treatment.

## Author Contributions

A-DW and Y-QL designed the study and approved the final version of the manuscript. WZ, JW and YQ performed the experiments. WZ and YB analyzed the data and wrote the manuscript. M-YL, J-WW, NJ and TC provided statistical analysis assistance and helped to revised the manuscript.

## Conflict of Interest Statement

The authors declare that the research was conducted in the absence of any commercial or financial relationships that could be construed as a potential conflict of interest.

## Abbreviations

8-OaS: 8-O-acetyl Shanzhiside methylester
ANOVA: analysis of variance
AUCs: area under the time-course curves
DMSO: dimethyl sulfoxide
ECL: enhanced chemiluminescence
ERK: extracellular regulated protein kinases
GFAP: glial fibrillary acidic protein
HRP: horseradish peroxidase
IGLR: Iridoid glycosides of L. rotate
IL-1β: interleukin-1 beta
JNK: Jun N-terminal kinase
L. rotata: Lamiophlomis rotata
MAPK: mitogen-activated protein kinase
NMDAR: N-methyl-D-aspartic acid receptor
NP: neuropathic pain
PB: phosphate buffer
PBS: phosphate buffer solution
PD98059: 2-(2-amino-3-methoxyphenyl)-4H-1-benzopyran-4-one (an ERK inhibitor)
pERK: phosphorylated extracellular regulated protein kinases
POD: post-operation day
PVDF: polyvinylidene difluoride
PWT: paw withdrawal threshold
RT: room temperature
SDS: sodium dodecyl sulfate
SEM: standard errors means
SM: shanzhiside methylester
SNL: spinal nerve ligation
TBS-T: tris-buffered saline with 0.02% Tween-20
TNF-α: tumor necrosis factor-alpha.
